# Auranofin Ameliorates Gouty Inflammation by Suppressing NLRP3 Activation and Neutrophil Migration via the IL-33/ST2–CXCL1 Axis

**DOI:** 10.3390/cells14191541

**Published:** 2025-10-02

**Authors:** Hyeyeon Yoo, Ahyoung Choi, Minjun Kim, Yongseok Gye, Hyeonju Jo, Seung-Ki Kwok, Youngjae Park, Jennifer Jooha Lee

**Affiliations:** 1 The Rheumatism Research Center, Catholic Research Institute of Medical Science, College of Medicine, The Catholic University of Korea, Seoul, Republic of Korea; yoohyeyeon@naver.com (H.Y.); wcay19@gmail.com (A.C.); rlaalswns102@naver.com (M.K.); yoogyoog@naver.com (Y.G.); hyeonju_jo@naver.com (H.J.); seungki73@catholic.ac.kr (S.-K.K.); 2 Department of Medical Sciences, College of Medicine, The Catholic University of Korea, Seoul, Republic of Korea; 3 Division of Rheumatology, Department of Internal Medicine, Seoul St. Mary’s Hospital, College of Medicine, The Catholic University of Korea, Seoul, Republic of Korea; elwin84@catholic.ac.kr

**Keywords:** gout, auranofin, NLRP3, IL-33/ST2, CXCL1, neutrophil migration, MSU crystals

## Abstract

**Highlights:**

**What are the main findings?**

- Auranofin suppresses NLRP3 inflammasome activation and attenuates the IL-33/ST2–CXCL1 axis, thereby reducing neutrophil recruitment in MSU-based models.
- Prophylactic dosing mitigates paw and air-pouch inflammation; enforced IL-33 overexpression abrogates these effects, indicating pathway dependency.

**What is the implication of the main finding?**

- Redox/thioredoxin-reductase targeting offers a dual-action strategy complementary to selective NLRP3 and CXCR2 blockade.
- The results motivate on-flare dosing and combination regimens (e.g., IL-33/ST2 or CXCR2 inhibition) under clinically aligned, exposure-matched designs.

**Abstract:**

Gout is a form of sterile inflammatory arthritis in which monosodium urate (MSU) crystals deposit and provoke a neutrophil-predominant response, primarily driven by activation of the NACHT, leucine-rich repeat, and pyrin domain-containing protein 3 (NLRP3) inflammasome. Here, we show that auranofin, a Food and Drug Administration (FDA)-approved anti-rheumatic agent, exerts anti-inflammatory effects in both in vitro and in vivo models of gout. Auranofin inhibited NLRP3 inflammasome activation in human THP-1 cells and murine macrophages, leading to reduced cleavage of caspase-1, interleukin-1β (IL-1β), and interleukin-18 (IL-18). In MSU crystal-induced mouse models, auranofin treatment reduced paw swelling, serum cytokine levels, and tissue inflammation. Notably, auranofin suppressed neutrophil migration and decreased expression of C-X-C motif chemokine ligand 1 (CXCL1) in inflamed foot tissue and air-pouch exudates. Mechanistically, auranofin disrupted the interleukin-33 (IL-33)/suppression of tumorigenicity 2 (ST2) axis, a key signaling pathway promoting neutrophil recruitment. Overexpression of IL-33 abolished the anti-inflammatory effects of auranofin, highlighting the central role of IL-33 in gout pathogenesis. Together, our findings suggest that auranofin alleviates MSU-induced inflammation by concurrently inhibiting NLRP3 inflammasome activation and IL-33-mediated neutrophil recruitment, supporting its potential as a dual-action therapeutic candidate for gout.

## 1. Introduction

Gout is an autoinflammatory arthritis driven by deposits of monosodium urate (MSU) crystals within peri-articular and intra-articular tissues, leading to abrupt, painful flares and functional impairment. These deposits precipitate abrupt, painful flares and contribute to a growing disease burden; recent Korean and broader Asian data indicate a continued rise in prevalence [1,2].

The inflammatory cascade is initiated when macrophages engulf MSU crystals and assemble the cytosolic NLRP3 inflammasome, a principal driver of sterile inflammation [3,4]. Once assembled, the NLRP3 inflammasome activates caspase-1, which cleaves pro-IL-1β and pro-IL-18 into bioactive cytokines and escalates the ensuing inflammatory response [5].

Acute attacks are characterized by brisk neutrophil influx. Through degranulation, production of reactive oxygen species, and formation of neutrophil extracellular traps (NETs), neutrophils sustain tissue damage and propagate the flare [6,7]. Among upstream signals that shape these responses, the alarmin interleukin-33 (IL-33) has emerged as a key modulator in gout; recent single-cell datasets also highlight IL-1β-driven pathways alongside dynamic changes in myeloid and regulatory T-cell compartments during flares [8,9,10].

IL-33 signals via its receptor ST2 on myeloid cells and promotes production of C-X-C motif chemokine ligand 1 (CXCL1), which engages CXCR2 to direct neutrophil trafficking [11,12]. In experimental gout, IL-33 augments CXCL1 from macrophages and stromal cells, whereas genetic or pharmacologic interruption of the IL-33/ST2 axis curtails CXCL1 levels and limits neutrophil accumulation [9,13,14]. These observations support a feed-forward loop in which MSU-triggered IL-33 reinforces CXCL1-dependent neutrophil recruitment and sustains inflammation. Together, these observations support a feed-forward loop in which MSU-triggered IL-33 reinforces CXCL1-dependent neutrophil recruitment and sustains inflammation, thereby positioning the IL-33/ST2–CXCL1 axis as a disease-proximal node integrating crystal sensing with early neutrophil trafficking [15,16,17].

Despite effective anti-inflammatory options such as colchicine, there remains a therapeutic gap: current agents do not concurrently blunt macrophage inflammasome activation and IL-33/CXCL1-driven neutrophil chemotaxis [18]. Auranofin, an FDA-approved oral gold(I) agent for rheumatoid arthritis, has been reported to temper inflammatory signaling by inhibiting nuclear factor kappa B (NF-κB) priming and activation of NLRP3 in diverse models [19,20], but its relevance to the IL-33/ST2/CXCL1 pathway in gout remains unclear.

Here, we investigate whether auranofin mitigates MSU-evoked inflammation by jointly limiting NLRP3 activation and IL-33/ST2-mediated neutrophil recruitment.

## 2. Materials and Methods

### 2.1. Animals

Male C57BL/6 mice (7 weeks old; OrientBio, Seongnam, Republic of Korea) were housed under specific pathogen-free (SPF) conditions. All animal procedures were approved by the Institutional Animal Care and Use Committee of the Catholic University of Korea (approval no. 2024–0007–02), and all animal experiments were conducted in accordance with institutional guidelines for animal welfare. All animal experiments were performed with at least *n* = 8 mice per group, and each experiment was independently repeated at least three times.

### 2.2. THP-1 Cell Culture and Stimulation

Human THP-1 cells (ATCC TIB-202) were cultured in RPMI-1640 medium supplemented with 10% fetal bovine serum (FBS) and 1% penicillin–streptomycin. Differentiation into macrophage-like cells was induced using 100 nM phorbol 12-myristate 13-acetate (PMA; Sigma-Aldrich, Busan, Republic of Korea) for 24 h, followed by a 24 h rest in PMA-free medium. After a 2 h priming with LPS (100 ng/mL; Sigma-Aldrich), cells were subsequently stimulated with 200 µg/mL monosodium urate (MSU) crystals (InvivoGen, San Diego, CA, USA) for 6 h [6,21,22]. These PMA-differentiation and LPS/MSU-stimulation conditions followed established THP-1 macrophage protocols [23,24]. Auranofin (0.005 or 0.5 µM; Sigma-Aldrich) was added 2 h prior to MSU stimulation [25,26,27,28,29]. At the concentrations tested, auranofin did not affect cell viability, as measured by the MTT assay (Supplementary Figure S1). All in vitro experiments were independently repeated five times.

### 2.3. Preparation and Culture of Mouse Bone Marrow-Derived Macrophages

BMDMs were generated from the femurs and tibiae of 6–8-week-old C57BL/6 mice. Bone marrow cells were flushed using sterile RPMI-1640, filtered through a 70 µm strainer, and erythrocytes were lysed using ACK buffer (Gibco, Grand Island, NY, USA, #A1049201). Cells were plated in Petri dishes and cultured in DMEM supplemented with 10% FBS, 1% penicillin–streptomycin, and 20 ng/mL M-CSF (Thermo Fisher, Waltham, MA, USA, #PMC2044). Medium was partially replaced on days 3 and 5. On day 7, cells were harvested. After a 4 h priming with LPS (100 ng/mL), BMDMs were exposed to MSU crystals (100 µg/mL) for 6 h. Auranofin (0.005 or 0.5 µM) was administered 2 h before MSU stimulation. These procedures followed standard M-CSF-driven BMDM generation protocols [30]. All in vitro experiments were independently repeated five times.

### 2.4. Primary Neutrophil Isolation

Neutrophils were isolated from the femurs and tibiae of C57BL/6 mice following a modified protocol [11]. After red blood cell lysis with ACK buffer, cells were layered on Histopaque-1119 (bottom) and Histopaque-1077 (top) (Sigma-Aldrich) and centrifuged (700× *g*, 30 min, room temperature, no brake). Neutrophils were collected from the interface, washed, and resuspended in complete RPMI medium [31].

### 2.5. IL-33 Overexpression

THP-1 and RAW264.7 cells were transiently transfected with species-specific IL-33 overexpression plasmid DNA using Lipofectamine 3000 (Invitrogen, Carlsbad, CA, USA); no viral transduction was performed. Human IL-33 plasmid (pLV[Exp]-EGFP:T2A:Puro-EF1A>hIL33[NM_001314044.1], VectorBuilder, Santa Clara, CA, USA, VB900020–4785ehb) or murine IL-33 plasmid (pLV[Exp]-Neo-EF1A>mIL33[NM_133775.3], VB900139–9123dqt). Respective empty plasmid vectors were used as mock controls. Transient transfections were performed per manufacturer guidance and published macrophage-transfection considerations [32,33]. Overexpression was validated by qPCR and Western blot.

### 2.6. Neutrophil Migration Assay

Human neutrophil migration was assessed using dHL-60 cells (differentiated in RPMI with 1% DMSO for 5 days) in 24-well Transwell plates with 5 μm pore inserts (Corning Inc., Corning, NY, USA). Chemoattractants in the lower chamber were supernatants from THP-1 cells stimulated with MSU with or without auranofin. After seeding, dHL-60 cells (1 × 10^6^ per well) were placed in the upper chamber in serum-free RPMI. In addition, Reparixin (10 μM) was used to assess the involvement of CXCL1 signaling in neutrophil chemotaxis. After 18 h at 37 °C, migrated cells were counted using Trypan blue and a hemocytometer.

Mouse neutrophil migration was performed using 24-well Transwell plates with 3 μm pores. Isolated neutrophils (1 × 10^6^/well) were seeded in the upper chamber, with chemoattractants in the lower chamber as described. After 18 h of incubation, cells were stained with Trypan blue and quantified. Transwell chemotaxis was conducted using a Boyden-type chamber protocol [34]; CXCR1/2 pathway inhibition was benchmarked as reviewed in [13].

### 2.7. Gouty Arthritis Model

Mice received daily intraperitoneal injection of auranofin (10 or 20 mg/kg) or saline for 5 weeks [25,26,35,36,37]. Subsequently, MSU crystals (300 μg in 0.1 mL PBS) were injected into the right hind paw as previously described [34]. Colchicine (1 mg/kg) was administered intraperitoneally 1.5 h before injection of MSU crystals. Paw thickness was measured both pre-injection and 24 h post-injection. Paws were collected for histological, protein, and gene expression analyses.

### 2.8. Air-Pouch Model

Mice received daily auranofin for 5 weeks. On week 4, 3 mL of sterile air was injected subcutaneously into the dorsal region on day 0 and day 3 to generate an air pouch. On day 6, 300 μg MSU in 1 mL PBS was injected into the pouch as previously described [38]. Colchicine (1 mg/kg) was administered intraperitoneally before MSU crystals injection. After 6 h, exudates were harvested.

### 2.9. Flow Cytometry

Cells from air-pouch exudates were stained with anti-CD11b-PE (clone M1/70, BioLegend, San Diego, CA, USA) and anti-Ly-6G-APC (clone 1A8, BioLegend), analyzed on a BD Fortessa, and evaluated using FlowJo software (FlowJo v10.10.0), with data acquisition and analysis performed in accordance with Current Protocols in Immunology [39].

### 2.10. Histology and Immunohistochemistry

Paraffin-embedded tissue sections (4 μm) were stained with hematoxylin and eosin (H&E) or subjected to immunohistochemistry using anti-IL-33 antibody (Abcam, Cambridge, UK). Antigen retrieval was performed using a pressure cooker and Dako solution. Detection was performed using the VECTASTAIN ABC kit (Vector Laboratories, Inc., Burlingame, CA, USA), DAB substrate, and hematoxylin counterstaining.

### 2.11. Western Blot Analysis

Proteins were extracted using RIPA buffer (Thermo Fisher) supplemented with protease and phosphatase inhibitors, quantified with the Bradford assay (Bio-Rad, Bio-Rad Laboratories, Hercules, CA, USA,), and resolved by SDS–PAGE and electroblotted to PVDF. The blots were blocked and then probed with primary antibodies: anti-cleaved IL-1β (mouse) polyclonal antibody (Proteintech, Rosemont, IL, USA, #PD039); anti-caspase-1 (pro/p10/p12) antibody (Abcam, #ab179515); anti-cleaved GSDMD N-terminal antibody (Abcam, #ab215203); anti-ASC polyclonal antibody (AdipoGen, Liestal, Switzerland, #AG-25B-0006-C100); anti-NLRP3 (D4D8T) rabbit monoclonal antibody (Cell Signaling Technology, Danvers, MA, USA, #15101S); anti-TNF-α antibody (Cell Signaling Technology, #3707S); anti-IL-18 antibody (Proteintech, #PA576082); anti-CXCL1/GROα antibody (Abcam, #ab86436); anti-MyD88 polyclonal antibody (eBioscience, San Diego, CA, USA, #14–6223–63); anti-phospho-NF-κB p65 (Ser536) antibody (Cell Signaling Technology, #3033S); anti-IL-33 antibody (Proteintech, #PA547007); anti-phosphor-IκBα antibody (MA5–15087); anti-GAPDH (clone EPR16891, Abcam, #ab181602); and β-Actin mouse monoclonal antibody (clone AC004, ABclonal, Woburn, MA, USA, #AC004). Membranes were then incubated with HRP-conjugated goat anti-mouse IgG (H + L) polyclonal antibody (Invitrogen, #626520, 1:2000) or HRP-conjugated goat anti-rabbit IgG (H + L) polyclonal antibody (Invitrogen, #31460, 1:5000) as secondary antibodies. Signal was detected using ECL reagent (Thermo Fisher) and visualized with an Amersham Imager (GE).

### 2.12. Quantitative Real-Time PCR

Total RNA was extracted using RNAiso Plus (Takara, Kusatsu, Japan), reverse-transcribed using a cDNA synthesis kit (Roche, Basel, Switzerland), and amplified with SYBR Green Master Mix on a LightCycler 96 system (Roche). Expression levels were normalized to GAPDH. Primer sequences used for qPCR are listed in Supplementary Table S1.

### 2.13. ELISA

Levels of IL-1β, IL-18, IL-33, and CXCL1 in cell supernatants and serum samples were measured using commercial ELISA kits (R&D Systems, Inc., Minneapolis, MN, USA) according to the manufacturer’s protocols.

### 2.14. Myeloperoxidase (MPO) Assay

MPO activity in air-pouch lavage fluid was determined using a commercially available kit (Abcam, #ab105136).

### 2.15. Statistical Analysis

All results are expressed as mean ± standard error of the mean (SEM). Statistical analyses were performed using GraphPad Prism (version 10.0, GraphPad Software, San Diego, CA, USA). Differences among multiple groups were evaluated by one-way analysis of variance (ANOVA), followed by Tukey’s post hoc test. When only two groups were compared, an unpaired two-tailed Student’s *t*-test was used. Normality of the data distribution was assessed using the Shapiro–Wilk test, and non-parametric tests (Mann–Whitney U test) were applied when appropriate. A *p*-value < 0.05 was considered statistically significant.

## 3. Results

### 3.1. Auranofin Attenuates MSU-Induced NLRP3 Inflammasome Activation in Human and Murine Macrophages

**Figure 1:**
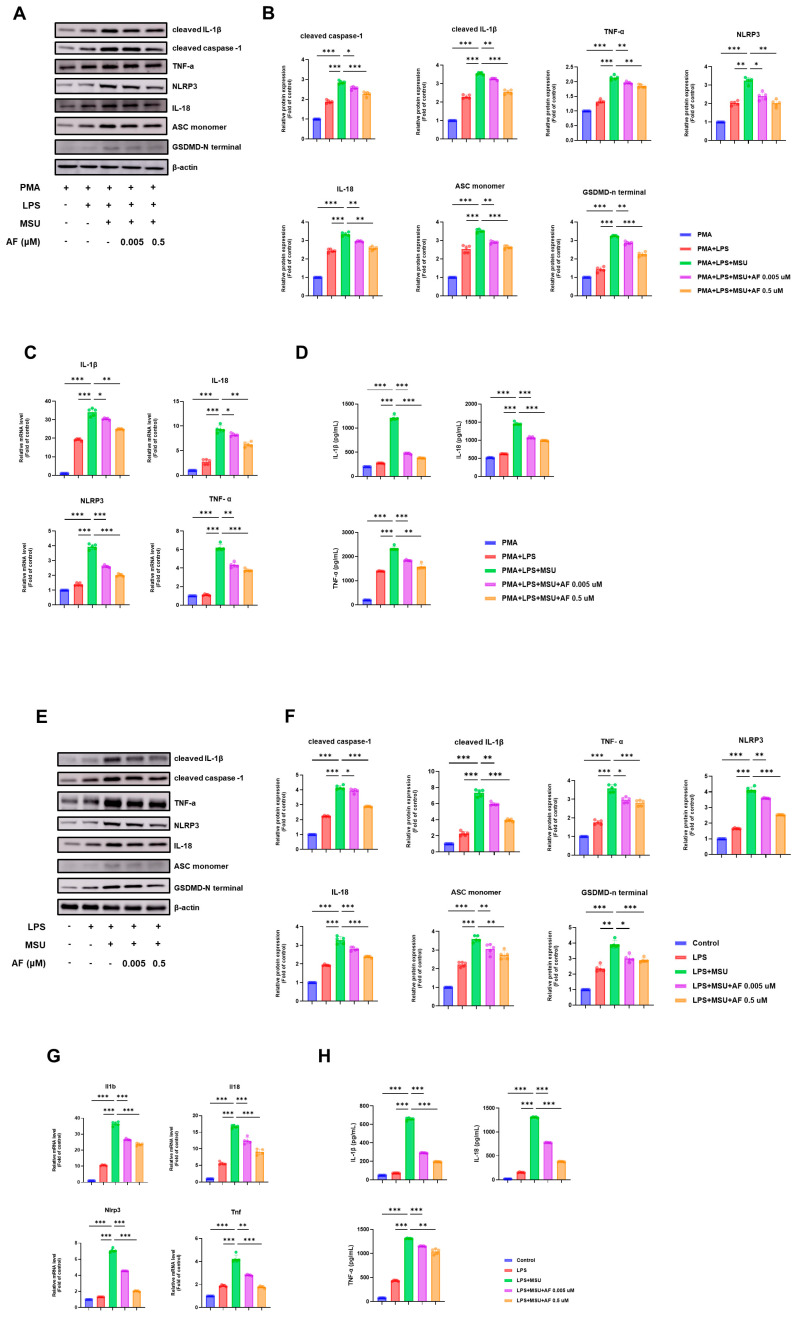
Auranofin attenuates MSU-induced NLRP3 inflammasome activation in human THP-1 macrophages and murine BMDMs. (**A**) Representative immunoblotting images of cleaved caspase-1, cleaved IL-1β, TNF-α, NLRP3, IL-18, ASC monomer, and GSDMD-N in THP-1 cells. (**B**) Densitometric quantification of immunoblotting data in THP-1 cells. (**C**) Relative mRNA expression of IL1B, IL18, TNF, and NLRP3 measured by qPCR in THP-1 cells. (**D**) ELISA results for secreted IL-1β, IL-18, and TNF-α in THP-1 culture supernatants. (**E**) Representative immunoblotting images in murine BMDMs. (**F**) Quantification of immunoblotting data in BMDMs. (**G**) Relative mRNA expression of Il1b, Il18, Tnf, and Nlrp3 in BMDMs. (**H**) ELISA results for IL-1β, IL-18, and TNF-α secretion in BMDMs. Data are presented as mean ± SEM from five independent experiments. * *p* < 0.05; ** *p* < 0.01; *** *p* < 0.001.

To evaluate whether auranofin suppresses MSU-induced activation of the NLRP3 inflammasome, we first examined human THP-1-derived macrophages. Stimulation with LPS and MSU significantly increased the protein levels of cleaved caspase-1, cleaved IL-1β, TNF-α, NLRP3, ASC monomer, IL-18, and GSDMD-N terminal, and these increases were markedly reduced following auranofin treatment, as confirmed by immunoblotting and densitometric quantification (Figure 1A,B). In addition, auranofin suppressed the mRNA expression of IL1B, IL18, TNF, and NLRP3 in a dose-dependent manner (Figure 1C) and attenuated the secretion of IL-1β, IL-18, and TNF-α, as determined by ELISA (Figure 1D).

Consistent findings were obtained in murine bone marrow-derived macrophages (BMDMs). Auranofin decreased MSU-induced expression of cleaved caspase-1, cleaved IL-1β, TNF-α, NLRP3, ASC monomer, IL-18, and GSDMD-N terminal, with quantification of band intensities confirming these reductions (Figure 1E,F). Furthermore, auranofin downregulated Il1b, Il18, Tnf, and Nlrp3 transcript levels (Figure 1G) and decreased the secretion of IL-1β, IL-18, and TNF-α, as measured by ELISA (Figure 1H). Collectively, these results indicate that auranofin effectively inhibits MSU-driven NLRP3 inflammasome activation and proinflammatory cytokine production in both human and murine macrophages.

### 3.2. Auranofin Alleviates MSU Crystal-Induced Inflammation in a Mouse Model of Acute Gouty Arthritis

**Figure 2:**
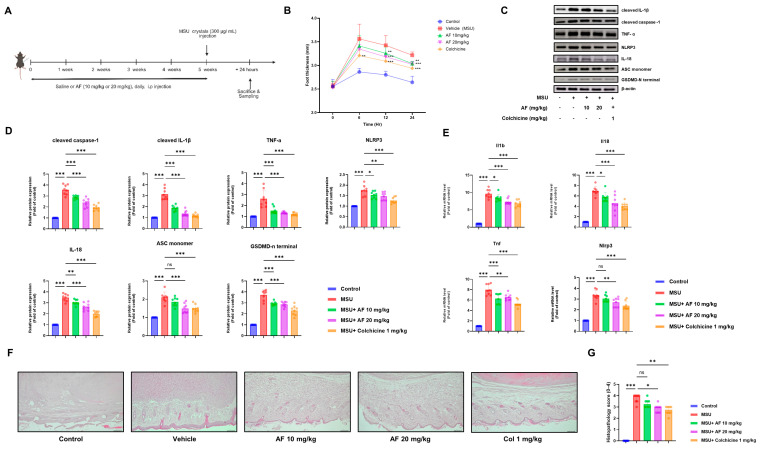
Auranofin alleviates MSU crystal-induced inflammation in a mouse model of acute gouty arthritis. (**A**) Experimental design showing daily intraperitoneal administration of auranofin (10 or 20 mg/kg) for five weeks prior to MSU crystal injection (300 µg/paw) into the hind paw. (**B**) Paw thickness was measured at the indicated time points following MSU injection. Auranofin significantly reduced MSU-induced swelling compared to vehicle treatment, with efficacy comparable to colchicine. (**C**) Representative immunoblots of cleaved caspase-1, cleaved IL-1β, TNF-α, NLRP3, IL-18, ASC monomer, and GSDMD-N terminal in paw tissues, with β-actin as a loading control. (**D**) Densitometric quantification of protein expression demonstrating significant attenuation of inflammasome activation and proinflammatory cytokine production by auranofin. (**E**) Relative mRNA expression of Il1b, Il18, Tnf, and Nlrp3 in paw tissues, as determined by qPCR. (**F**) Representative H&E-stained sections of paw tissues showing inflammatory cell infiltration and tissue damage. (**G**) Histopathological scores indicating reduced inflammation and improved tissue integrity in auranofin-treated groups. Data are presented as mean ± SEM (*n* = 8 per group). * *p* < 0.05, ** *p* < 0.01, *** *p* < 0.001 vs. MSU-treated group. ns, not significant.

To evaluate the anti-inflammatory effects of auranofin in vivo, mice were administered daily intraperitoneal injections of auranofin for five weeks prior to MSU crystal challenge (Figure 2A). Injection of MSU into the hind paw induced pronounced swelling, which was significantly attenuated in auranofin-treated animals in a dose-dependent manner, comparable to the effects of colchicine (Figure 2B).

At the molecular level, immunoblot analysis of paw tissues revealed robust induction of cleaved caspase-1, cleaved IL-1β, TNF-α, NLRP3, IL-18, ASC monomer, and GSDMD-N terminal following MSU injection (Figure 2C). These increases were markedly reduced by auranofin administration, as confirmed by densitometric quantification (Figure 2D). In line with the protein changes, quantitative PCR demonstrated that auranofin significantly decreased the MSU-induced upregulation of Il1b, Il18, Tnf, and Nlrp3 transcripts in paw tissues (Figure 2E).

Consistently, serum cytokine measurements by ELISA showed that auranofin suppressed circulating IL-1β and IL-18 levels compared to MSU-injected controls (Supplementary Figure S2A). Histopathological evaluation using H&E staining further demonstrated reduced inflammatory cell infiltration and tissue injury in auranofin-treated mice (Figure 2F), as was reflected by significantly improved histological scores (Figure 2G). Collectively, these results indicate that auranofin effectively mitigates MSU-driven inflammation in vivo by attenuating NLRP3 inflammasome activation, proinflammatory cytokine production, and associated joint pathology.

### 3.3. Auranofin Suppresses MSU Crystal-Induced Inflammation and Neutrophil Recruitment in a Mouse Air-Pouch Model

**Figure 3:**
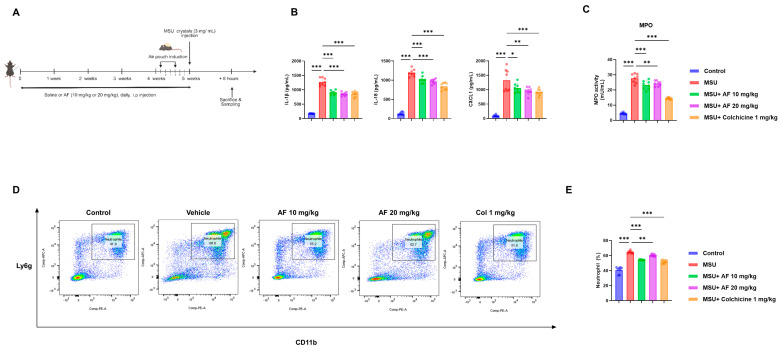
Auranofin suppresses MSU crystal-induced inflammation and neutrophil infiltration in a mouse air-pouch model. (**A**) Experimental timeline for the air-pouch model and auranofin treatment. (**B**) Concentrations of IL-1β, IL-18, TNF-α, and CXCL1 in air-pouch lavage fluid. (**C**) Myeloperoxidase (MPO) activity in lavage fluid. (**D**) Flow cytometric analysis of CD11b^+^Ly6G^+^ neutrophils in air-pouch exudates. (**E**) Quantification of CD11b^+^Ly6G^+^ cell populations in (**D**). Data represent mean ± SEM of at least three independent experiments. * *p* < 0.05; ** *p* < 0.01; *** *p* < 0.001.

We next examined auranofin in vivo using a mouse air-pouch gout model to further characterize its anti-inflammatory effects. As illustrated in the experimental scheme (Figure 3A), auranofin was administered daily for 5 weeks prior to MSU crystal injection. Auranofin treatment significantly reduced the levels of IL-1β and IL-18 in serum compared to MSU-treated controls (Supplementary Figure S2B).

In air-pouch lavage fluid, auranofin markedly decreased the concentrations of IL-1β, IL-18, TNF-α, and CXCL1, a major neutrophil chemoattractant (Figure 3B). In addition, myeloperoxidase (MPO) activity—a marker of neutrophil activation—was significantly reduced following auranofin treatment (Figure 3C).

Flow cytometric analysis revealed that the proportion of CD11b^+^Ly6G^+^ neutrophils in the air pouch was substantially decreased in auranofin-treated mice (Figure 3D), with quantitative analysis confirming this reduction (Figure 3E).

Taken together, the data show that auranofin curtails MSU-evoked cytokine output and limits neutrophil influx in vivo.

### 3.4. Auranofin Suppresses MSU Crystal-Induced Neutrophil Migration by Inhibiting CXCL1 Production

**Figure 4:**
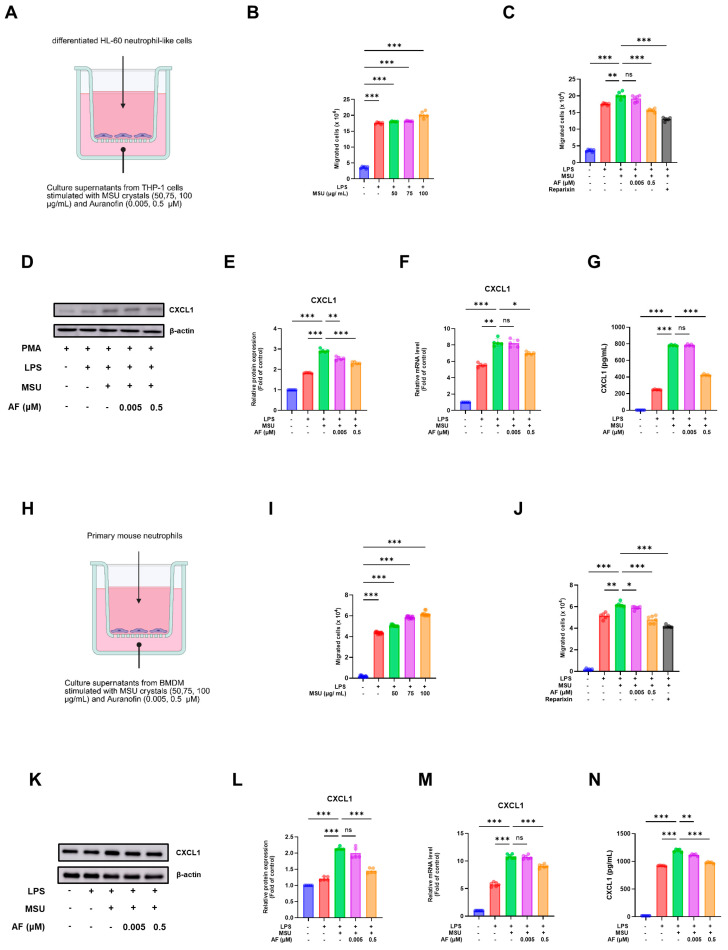
Auranofin inhibits MSU crystal-induced neutrophil migration by suppressing CXCL1 production. (**A**) Schematic diagram of the Transwell migration assay using dHL-60 cells and THP-1 cell supernatants as chemoattractants. (**B**) Migration of dHL-60 cells in response to increasing concentrations of MSU (50, 75, and 100 μg/mL). (**C**) Migration of dHL-60 cells in response to MSU (100 μg/mL) with or without auranofin (0.005 or 0.5 μM) or Reparixin (10 μM). (**D**) Representative immunoblotting image of CXCL1 protein levels in THP-1 cells. (**E**) Densitometric quantification of CXCL1 protein expression in (**D**). (**F**) Relative mRNA expression of CXCL1 in THP-1 cells. (**G**) CXCL1 concentrations in THP-1 supernatants measured by ELISA. (**H**) Schematic diagram of the Transwell migration assay using primary murine neutrophils and BMDM supernatants. (**I**–**N**) Migration, protein, mRNA, and ELISA analyses of CXCL1 in murine cells, performed as described in (**B**–**G**). Data represent mean ± SEM of three independent experiments. * *p* < 0.05; ** *p* < 0.01; *** *p* < 0.001.

We assessed neutrophil chemotaxis in a Transwell system by seeding dHL-60 cells in the upper chamber and using THP-1 supernatants (with or without auranofin) as chemoattractants in the lower chamber. Supernatants from MSU-stimulated THP-1 cells, with or without auranofin treatment, were placed in the lower chamber as chemoattractants, while dHL-60 cells were placed in the upper chamber (Figure 4A).

To optimize the MSU concentration for chemotaxis, dHL-60 cells were exposed to varying doses of MSU (50, 75, and 100 µg/mL). Neutrophil migration increased in a dose-dependent manner, peaking at 100 μg/mL (Figure 4B). Using this concentration, we evaluated the effect of auranofin (0.005 or 0.5 µM) and Reparixin (10 µM), a CXCR2 antagonist. Both agents significantly reduced the number of migrated cells (Figure 4C), as was confirmed by Gram staining and quantification (Supplementary Figure S3A,B).

To elucidate the mechanism underlying this effect, we analyzed the expression of CXCL1, a key neutrophil-attracting chemokine. Western blot analysis showed that auranofin suppressed MSU-induced CXCL1 expression in THP-1 cells in a dose-dependent manner (Figure 4D), and band quantification confirmed this result (Figure 4E). Consistently, CXCL1 mRNA levels (Figure 4F) and protein concentrations in cell culture supernatants (Figure 4G) were markedly reduced following auranofin treatment.

To validate these findings in murine cells, supernatants from MSU-stimulated BMDMs were used as chemoattractants in a Transwell migration assay with primary mouse neutrophils (Figure 4H). Auranofin-treated BMDMs exhibited reduced chemotactic activity, as indicated by decreased numbers of migrated neutrophils (Figure 4I,J). In parallel, auranofin diminished CXCL1 expression at both protein and mRNA levels, as shown by immunoblotting, ELISA, and qPCR (Figure 4K–N).

Collectively, these data demonstrate that auranofin attenuates MSU crystal-induced neutrophil recruitment by inhibiting CXCL1 production in both human and murine macrophages.

### 3.5. Auranofin Suppresses IL-33-Induced Proinflammatory Signaling and Neutrophil Chemotaxis In Vitro

IL-33, a member of the IL-1 cytokine family, is known to amplify inflammation by promoting neutrophil recruitment and activation. Its expression is elevated in the synovial fluid and serum of patients with gout and in murine models of MSU crystal-induced gouty arthritis. Mechanistically, IL-33 enhances neutrophilic infiltration by inducing the production of chemokines such as CXCL1 from macrophages and stromal cells, thereby sustaining sterile inflammatory responses.

**Figure 5:**
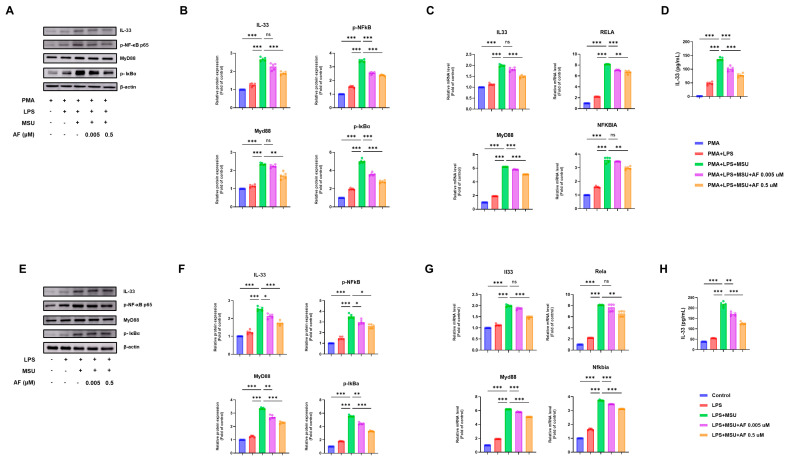
Auranofin suppresses neutrophilic inflammation by inhibiting the IL-33/ST2 signaling pathway. (**A**) Immunoblot analysis IL-33, phosphorylated NF-κB p65, MyD88, and phosphorylated IκBα in MSU-stimulated THP-1 cells with or without auranofin. (**B**) Densitometric quantification of immunoblotting data in THP-1 cells. (**C**) Relative mRNA expression levels of IL33, NFKBIA (encoding IκBα), MYD88, and RELA (encoding NF-κB p65) in THP-1 cells. (**D**) IL-33 concentrations in THP-1 supernatants measured by ELISA. (**E**) Immunoblot analysis of IL-33, phosphorylated NF-κB p65, MyD88, and phosphorylated IκBα in MSU-stimulated BMDMs with or without auranofin. (**F**) Densitometric quantification of immunoblotting data in BMDMs. (**G**) Relative mRNA expression levels of Il33, Nfkbia, Myd88, and Rela in BMDMs. (**H**) IL-33 concentrations in BMDM supernatants measured by ELISA. Data represent mean ± SEM of five independent experiments. * *p* < 0.05; ** *p* < 0.01; *** *p* < 0.001. ns, not significant.

To determine whether auranofin modulates IL-33 signaling during MSU crystal-induced inflammation, we assessed the expression of IL-33 and downstream components of the ST2 signaling cascade in human THP-1 cells. Auranofin treatment markedly attenuated MSU-induced upregulation of IL-33, phosphorylated NF-κB p65, MyD88, and phosphorylated IκBα (Figure 5A), as confirmed by band intensity quantification (Figure 5B). These changes were accompanied by significant reductions in mRNA levels of IL33, RELA (encoding NF-κB p65), MYD88, and NFKBIA (encoding IκBα) in a dose-dependent manner (Figure 5C). Consistent with these observations, ELISA revealed reduced IL-33 secretion in auranofin-treated cells (Figure 5D).

We next evaluated whether these effects were recapitulated in murine BMDMs. Auranofin suppressed the MSU crystal-induced expression of IL-33, phosphorylated NF-κB p65, MyD88, and phosphorylated IκBα (Figure 5E), with corresponding decreases validated by quantitative analysis (Figure 5F). Similarly, auranofin reduced the mRNA expression of Il33, Rela, Myd88, and Nfkbia (Figure 5G), and IL-33 secretion into the culture supernatant was significantly diminished in a dose-dependent fashion (Figure 5H).

Collectively, these findings demonstrate that auranofin inhibits MSU crystal-induced IL-33/ST2 signaling and downstream inflammatory responses in both human and murine macrophages.

### 3.6. Auranofin Attenuates IL-33-Mediated Inflammation In Vivo Through Suppression of the IL-33/ST2 Signaling Axis

**Figure 6:**
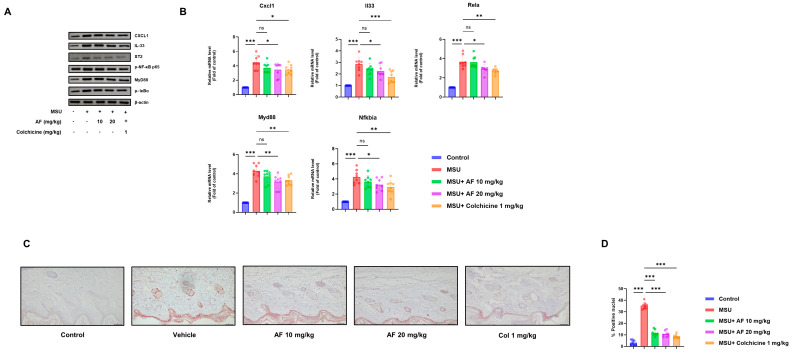
Auranofin attenuates IL-33-mediated inflammation in vivo through suppression of the IL-33/ST2 signaling axis. (**A**) Immunoblot analysis of CXCL1, IL-33, ST2, phosphorylated NF-κB p65 (p-p65), MyD88, and phosphorylated IκBα in paw tissues from MSU-injected mice treated with or without auranofin. (**B**) Relative mRNA expression levels of Cxcl1, Il33, Rela, Myd88, and Nfkbia in paw tissues. (**C**) Representative images of IL-33 immunohistochemical staining in the epidermal region of mouse foot tissue. (**D**) Quantification of IL-33-positive signal shown in (**C**). Data represent mean ± SEM. * *p* < 0.05; ** *p* < 0.01; *** *p* < 0.001; ns, not significant.

To determine whether auranofin modulates IL-33/ST2 signaling in vivo, we analyzed inflamed foot tissue from MSU-injected mice. Auranofin treatment significantly reduced protein levels of CXCL1, IL-33, ST2, phospho-NF-κB p65 (p-p65), MyD88, and phospho-IκBα, as shown by immunoblotting (Figure 6A), with corresponding reductions confirmed by densitometric analysis (Supplementary Figure S4). In parallel, auranofin-treated mice exhibited decreased mRNA expression of Cxcl1, Il33, Rela (encoding NF-κB p65) Myd88, and Nfkbia (Figure 6B).

IL-33 is known to be upregulated during gouty inflammation and is primarily expressed by epithelial and endothelial cells in inflamed tissues. Based on this, we performed immunohistochemical analysis of the epidermal region in foot tissue. IL-33 expression was markedly lower in the auranofin-treated group compared with MSU-injected controls (Figure 6C), and this reduction was further supported by quantitative scoring (Figure 6D).

### 3.7. IL-33 Overexpression Overrides the Anti-Inflammatory Effects of Auranofin in Human and Murine Macrophages

**Figure 7:**
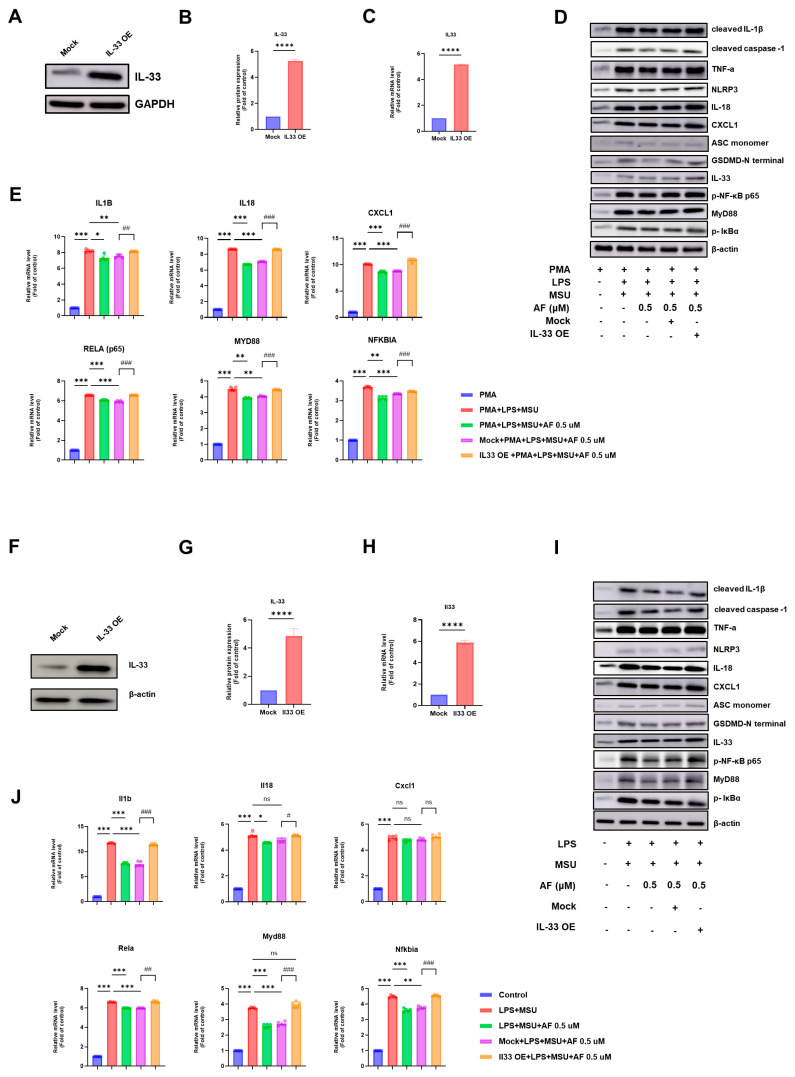
IL-33 overexpression overrides the anti-inflammatory effects of auranofin in human and murine macrophages. (**A**) Immunoblot analysis of IL-33 protein levels in mock- and IL-33-transfected THP-1 cells. (**B**) Densitometric quantification of IL-33 expression shown in A. (**C**) Relative mRNA expression of IL33 in mock- and IL-33-transfected THP-1 cells. (**D**) Representative immunoblotting images of cleaved IL-1β, cleaved caspase-1, TNF-α, NLRP3, IL-18, CXCL1, ASC monomer, GSDMD-N, IL-33, phosphorylated NF-κB p65 (p-p65), MyD88, and phosphorylated IκBα in THP-1 cells treated under five conditions: (1) PMA only; (2) PMA + LPS + MSU; (3) PMA + LPS + MSU + auranofin; (4) IL-33 mock + PMA + LPS + MSU + auranofin; and (5) IL-33 overexpression + PMA + LPS + MSU + auranofin. (**E**) Relative mRNA expression of IL1B, IL18, CXCL1, RELA (encoding NF-κB p65), MYD88, and NFKBIA (encoding IκBα) in THP-1 cells treated, as in (**D**). (**F**) Representative immunoblotting images of IL-33 protein levels in mock- and IL-33-transfected RAW264.7 cells. (**G**) Densitometric quantification of IL-33 expression shown in (**A**). (**H**) Relative mRNA expression of IL33 in mock- and IL-33-transfected THP-1 cells. (**I**) Immunoblot analysis of cleaved IL-1β, cleaved caspase-1, TNF-α, NLRP3, IL-18, CXCL1, ASC monomer, GSDMD-N, IL-33, phosphorylated NF-κB p65 (p-p65), MyD88, and phosphorylated IκBα in RAW264.7 cells treated under five conditions: (1) control; (2) LPS + MSU; (3) LPS + MSU + auranofin; (4) IL-33 mock + LPS + MSU + auranofin; and (5) IL-33 overexpression + LPS + MSU + auranofin. (**J**) Relative mRNA expression of Il1b, Il18, Cxcl1, Rela (encoding NF-κB p65), Myd88, and Nfkbia (encoding IκBα) in RAW264.7 cells treated, as in (**D**). Data represent mean ± SEM from at least five independent experiments. * *p* < 0.05, ** *p* < 0.01, *** *p* < 0.001, **** *p* < 0.0005, vs. MSU; # *p* < 0.05, ## *p* < 0.01, ### *p* < 0.001 vs. Mock; ns, not significant.

To determine whether the anti-inflammatory effects of auranofin are mediated through IL-33, we established an IL-33 overexpression model using THP-1 cells. IL-33-transfected cells exhibited significantly elevated IL-33 expression at both the protein and mRNA levels compared with mock-transfected controls, as demonstrated by Western blotting and qPCR (Figure 7A–C).

We next evaluated whether auranofin retained its anti-inflammatory activity in the setting of IL-33 overexpression. THP-1 cells were assigned to five experimental groups: (1) PMA only; (2) PMA + LPS + MSU; (3) PMA + LPS + MSU + auranofin (0.5 µM); (4) IL-33 mock + PMA + LPS + MSU + auranofin; and (5) IL-33 overexpression + PMA + LPS + MSU + auranofin. Auranofin treatment reduced the expression of cleaved IL-1β, cleaved caspase-1, TNF-α, IL-18, NLRP3, ASC, GSDMD-N, CXCL1, phospho-NF-κB p65, MyD88, and phospho-IκBα in group (3), but these inhibitory effects were abolished in IL-33-overexpressing cells (Figure 7D). Quantitative densitometric analysis confirmed this loss of suppression (Supplementary Figure S5A).

Similarly, auranofin decreased the mRNA expression of IL1B, IL18, CXCL1, RELA (encoding NF-κB p65), MYD88, and NFKBIA (encoding IκBα) in mock-transfected cells, but these reductions were not observed in IL-33-overexpressing cells (Figure 7E).

To confirm these findings in murine macrophages, we conducted parallel experiments in IL-33-overexpressing RAW264.7 cells, which allow for higher transfection efficiency than primary bone marrow-derived macrophages [40,41]. As in human cells, auranofin failed to suppress the expression of inflammatory mediators in IL-33-overexpressing murine macrophages (Figure 7F–J, Supplementary Figure S5B).

Collectively, these findings indicate that IL-33 overexpression negates the anti-inflammatory effects of auranofin, underscoring the importance of the IL-33/ST2 signaling axis in mediating auranofin’s immunomodulatory actions.

## 4. Discussion

Our findings identify auranofin as a dual-acting modulator of gouty inflammation. In human THP-1-derived macrophages and murine macrophages, auranofin dampened the NLRP3 cascade—evidenced by lower caspase-1 activity and reduced IL-1β, IL-18, and GSDMD-N—effects that were mirrored in vivo in paw and air-pouch models.

In vitro, auranofin decreased MSU-induced expression of inflammasome readouts and attenuated secretion of key cytokines, together with downregulation of Il1b/IL1B, Il18/IL18, Tnf/TNF, and Nlrp3/NLRP3 transcripts. These data are consistent with inhibition of NF-κB-dependent priming and redox-sensitive steps that license NLRP3 activity.

In vivo, prophylactic auranofin lessened paw swelling and improved histopathologic scores while lowering tissue and systemic cytokines. In the air-pouch model, auranofin reduced IL-1β, IL-18, TNF-α, and CXCL1 in exudates; decreased myeloperoxidase activity; and reduced the proportion of CD11b+Ly6G+ neutrophils, indicating effective control of neutrophil-dominant inflammation.

Mechanistically, auranofin suppressed the IL-33/ST2 axis: IL-33 protein and transcript were reduced alongside MyD88 and phosphorylated IκBα and NF-κB p65 in macrophages and inflamed tissues. This suggests disruption of a feed-forward circuit in which IL-33 amplifies CXCL1 production to drive neutrophil recruitment [11,12,13,14,18]. Accordingly, auranofin limited CXCL1 at mRNA and protein levels and restrained chemotaxis in Transwell assays; the CXCR2 antagonist Reparixin produced concordant effects [13]. Although neutrophils dominate the early cellular influx, tissue-resident macrophages and dendritic cells likely contribute to the initial cytokine–chemokine milieu and may serve as sources or amplifiers of IL-33 and CXCL1 in response to crystals and alarmins. Thus, part of auranofin’s activity may occur within these resident compartments [10,11,14,17].

A critical observation is that enforced IL-33 expression negated auranofin’s anti-inflammatory activity, positioning the IL-33/ST2 pathway as required for the drug’s benefit. This dependency integrates our data across models and explains the concurrent effects on inflammasome readouts and neutrophil trafficking. Future work will directly compare prophylactic and on-flare regimens and evaluate rational combinations in MSU-based models. We will also compare auranofin’s dual-action profile with next-generation NLRP3 inhibitors [19] (e.g., dapansutrile) to determine whether redox modulation confers additional benefits or risks. Specifically, we will test auranofin with IL-33/ST2 blockade or CXCR2 antagonism under conditions of experimentally elevated IL-33 signaling.

This study has limitations. We modeled acute MSU-driven inflammation in male C57BL/6 mice. Chronic and tophaceous disease, sex-specific effects, and pharmacokinetic-pharmacodynamic relationships were not addressed; accordingly, future studies will explicitly incorporate sex as a biological variable. In addition, pathways beyond IL-33—such as CCL2 or IL-6—may contribute to residual neutrophilia [13,14]. Given auranofin’s targeting of thioredoxin reductase and redox-sensitive signaling, it may plausibly modulate IL-6, CCL2, and NETosis [26,29,41]. We therefore note this as a potential mechanism and will quantify IL-6 and CCL2 and assess NETosis alongside CXCL1 in follow-up experiments [8,42,43]. Separately, auranofin’s tolerability in gout requires careful consideration given historical gastrointestinal adverse events in rheumatoid arthritis; dose optimization or reformulation could improve the therapeutic window [20]. To better align with clinical constraints, we will conduct exposure-matching studies, administering auranofin orally with PK/PD sampling of circulating gold and thioredoxin-reductase inhibition [20,26,29,41]. We will also implement acute and post-flare therapeutic-timing designs.

Finally, our in vivo design used a pre-induction, once-daily auranofin regimen to model prophylactic/maintenance exposure, a paradigm commonly employed in MSU models and clinically analogous to anti-inflammatory prophylaxis when initiating urate-lowering therapy. While this provides mechanistic insight into whether sustained target engagement can blunt subsequent flare magnitude, post-induction dosing remains clinically important and will be evaluated in future studies that administer auranofin at the time of or after MSU challenge. Additionally, whether auranofin, in combination with established gout therapies such as colchicine or selective NLRP3 inhibitors, yields additive or synergistic effects has not been adequately investigated and warrants confirmation in dedicated follow-up studies. To strengthen causal inference at the pathway level, future studies will incorporate rescue designs that pair auranofin with IL-33/ST2 blockade—for example, neutralizing anti-IL-33 antibodies (tozorakimab/MEDI3506) [44,45] or ST2 inhibition via antibodies or soluble decoys (e.g., sST2-Fc)—under MSU conditions in vitro, as well as in vivo [11]. These experiments will test whether dampening IL-33/ST2 signaling restores or augments auranofin responsiveness under conditions of heightened IL-33 activity.

Overall, our results support repurposing auranofin for gout by simultaneously restraining NLRP3 signaling and IL-33/ST2–CXCL1-dependent neutrophil influx—two complementary nodes not targeted in combination by existing therapies.

## 5. Conclusions

Auranofin exerts dual anti-inflammatory actions in experimental gout by restraining NLRP3 inflammasome activity and limiting IL-33/ST2–CXCL1-driven neutrophil trafficking. Across human THP-1-derived and murine macrophages, and in vivo paw and air-pouch models, auranofin reduced caspase-1 activation and IL-1β/IL-18 release, decreased CXCL1, and curtailed neutrophil influx. Enforced IL-33 overexpression eliminated these benefits, underscoring dependency on the IL-33/ST2 pathway.

These data position auranofin’s redox/thioredoxin-reductase targeting as a mechanistically complementary approach to current and emerging gout therapeutics. Translational next steps include comparing prophylactic versus on-flare regimens, conducting exposure-matching studies with oral dosing and PK/PD assessment of circulating gold and thioredoxin-reductase inhibition, and testing rational combinations with IL-33/ST2 or CXCR2 blockade and with selective NLRP3 inhibitors.

## Data Availability

All data supporting the findings of this study are available from the corresponding author upon reasonable request.

## Supplementary Materials

The following supporting information can be downloaded at https://www.mdpi.com/article/10.3390/cells14191541/s1, Figure S1: Cytotoxicity of Auranofin in THP-1 cells and BMDMs assessed by MTT assay; Figure S2: Auranofin reduces serum IL-1β and IL-18 levels in MSU-induced gout models; Figure S3: Auranofin suppresses neutrophil migration in response to MSU stimulation; Figure S4: Densitometric analysis of IL-33/ST2 signaling components in inflamed foot tissues; Figure S5: Quantitative analysis of IL-33 overexpression effects in THP-1 and Raw 264.7 cells. Table S1. Primer sequences used for qPCR analysis.
