# Supplementary material for: Auranofin Ameliorates Gouty Inflammation by Suppressing NLRP3 Activation and Neutrophil Migration via the IL-33/ST2–CXCL1 Axis
Source: Cells. 2025 Oct 2;14(19):1541. doi: 10.3390/cells14191541 (PMC12523252; doi:10.3390/cells14191541)
Supplement: Supplementary file 1 [file cells-14-01541-s001.zip › cells-3894323-supplementary.pdf]

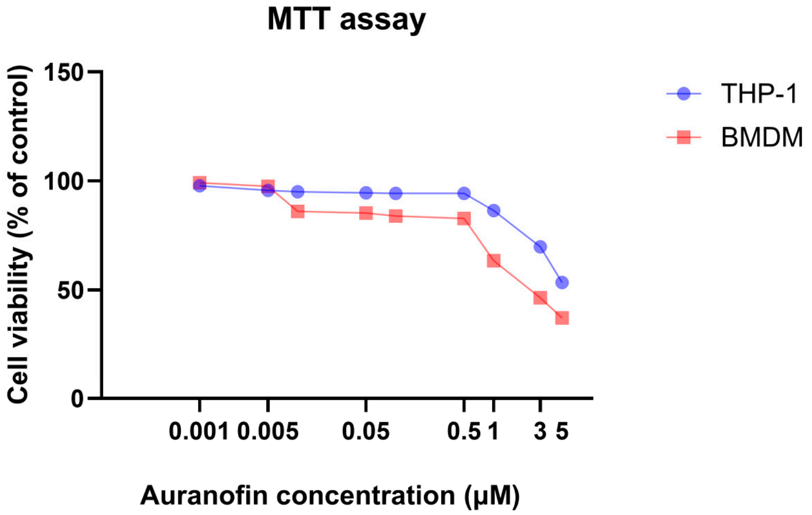


**Supplemental Figure 1. Cytotoxicity of Auranofin in THP-1 cells and BMDMs assessed by MTT assay.**

THP-1–derived macrophages and murine bone marrow–derived macrophages (BMDMs) were treated with increasing concentrations of Auranofin (0.001–5 μM) for 24 h, and cell viability was measured using the MTT assay. Data are expressed as the percentage of viable cells relative to untreated control cells. Auranofin at concentrations up to 0.5 μM did not significantly affect cell viability, whereas higher doses (≥1 μM) led to a dose-dependent reduction in viability in both cell types. All in vitro experiments were independently repeated five times.

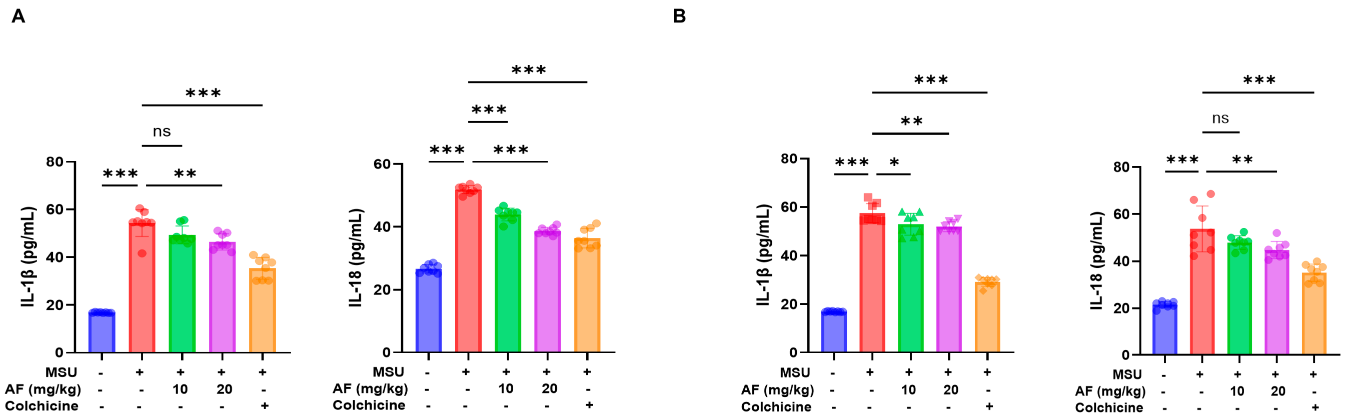


**Supplemental Figure 2. Auranofin reduces serum IL-1β and IL-18 levels in MSU-induced gout models.**

(A) Serum concentrations of IL-1β and IL-18 in mice subjected to MSU-induced gouty arthritis and treated with or without Auranofin.
(B) Serum levels of IL-1β and IL-18 measured in an air pouch model of MSU-induced inflammation. Cytokine levels were determined by ELISA. Data are presented as mean ± SEM (n = 8 per group). $^*P < 0.05$, $^{**}P < 0.01$, $^{***}P < 0.001$ by one-way ANOVA with Tukey's post hoc test.

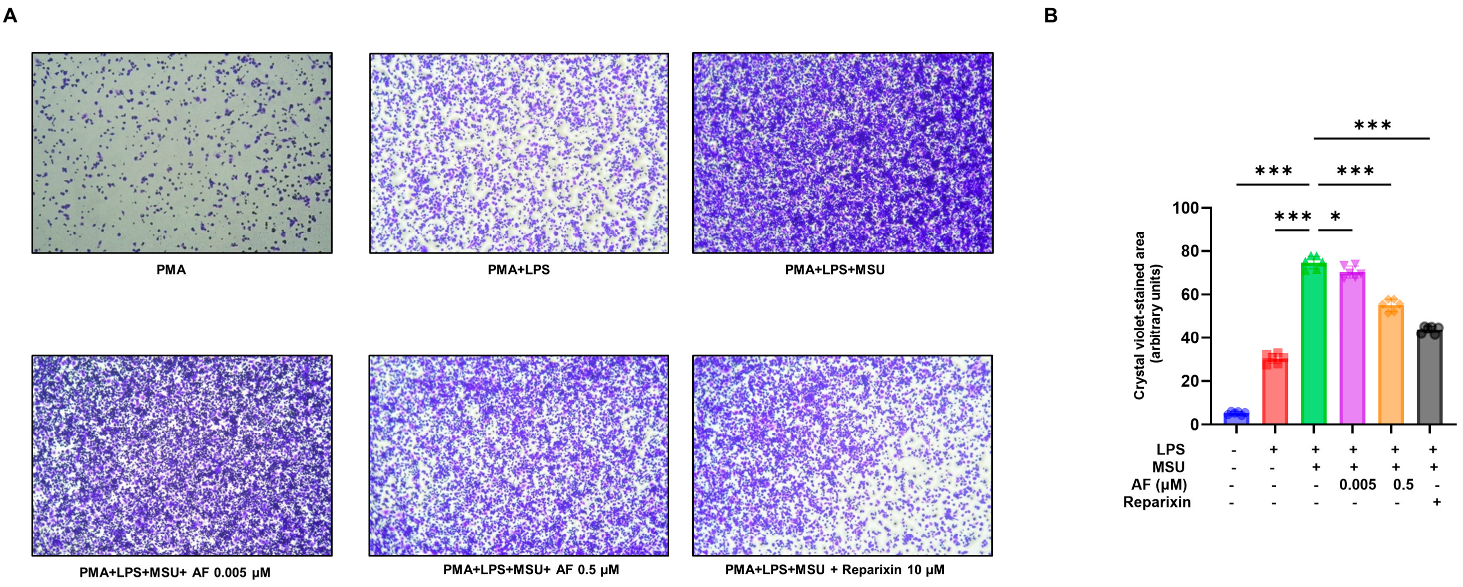


**Supplemental Figure 3. Auranofin suppresses neutrophil migration in response to MSU stimulation.**

(A) Representative Gram-stained images of dHL-60 cells that migrated through the Transwell membrane in response to MSU stimulation (100 μg/mL) and treatment with Auranofin (0.005 or 0.5 μM) or Reparixin (10 μM).
(B) Quantification of migrated dHL-60 cells under the same conditions. Data represent mean ± SEM of three independent experiments. *P < 0.05, **P < 0.01 by one-way ANOVA with Tukey's post hoc test.

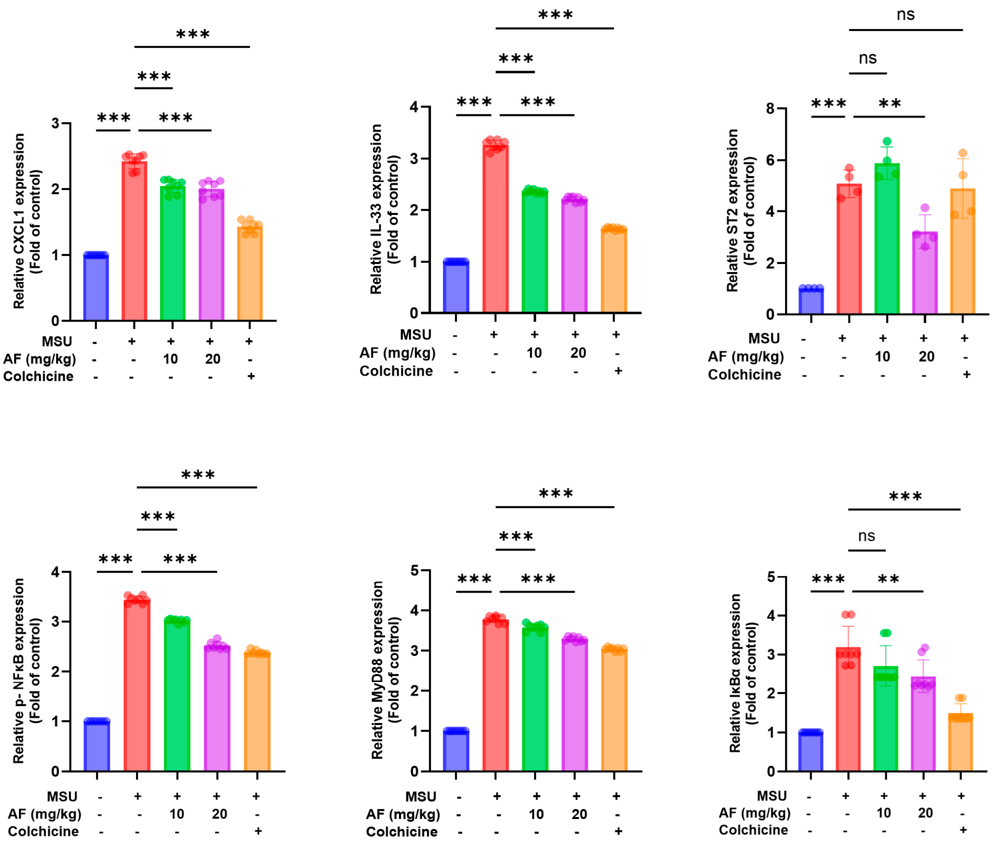


**Supplemental Figure 4. Densitometric analysis of IL-33/ST2 signaling components in inflamed foot tissues.**

Quantification of CXCL1, IL-33, ST2, phosphorylated NF-κB p65 (p-p65), MyD88 and phosphorylated IκBα protein levels in foot tissues from MSU-injected mice with or without Auranofin treatment. Data were normalized to β-actin and represent mean ± SEM (n = 8 per group). *P < 0.05, **P < 0.01.

**A**

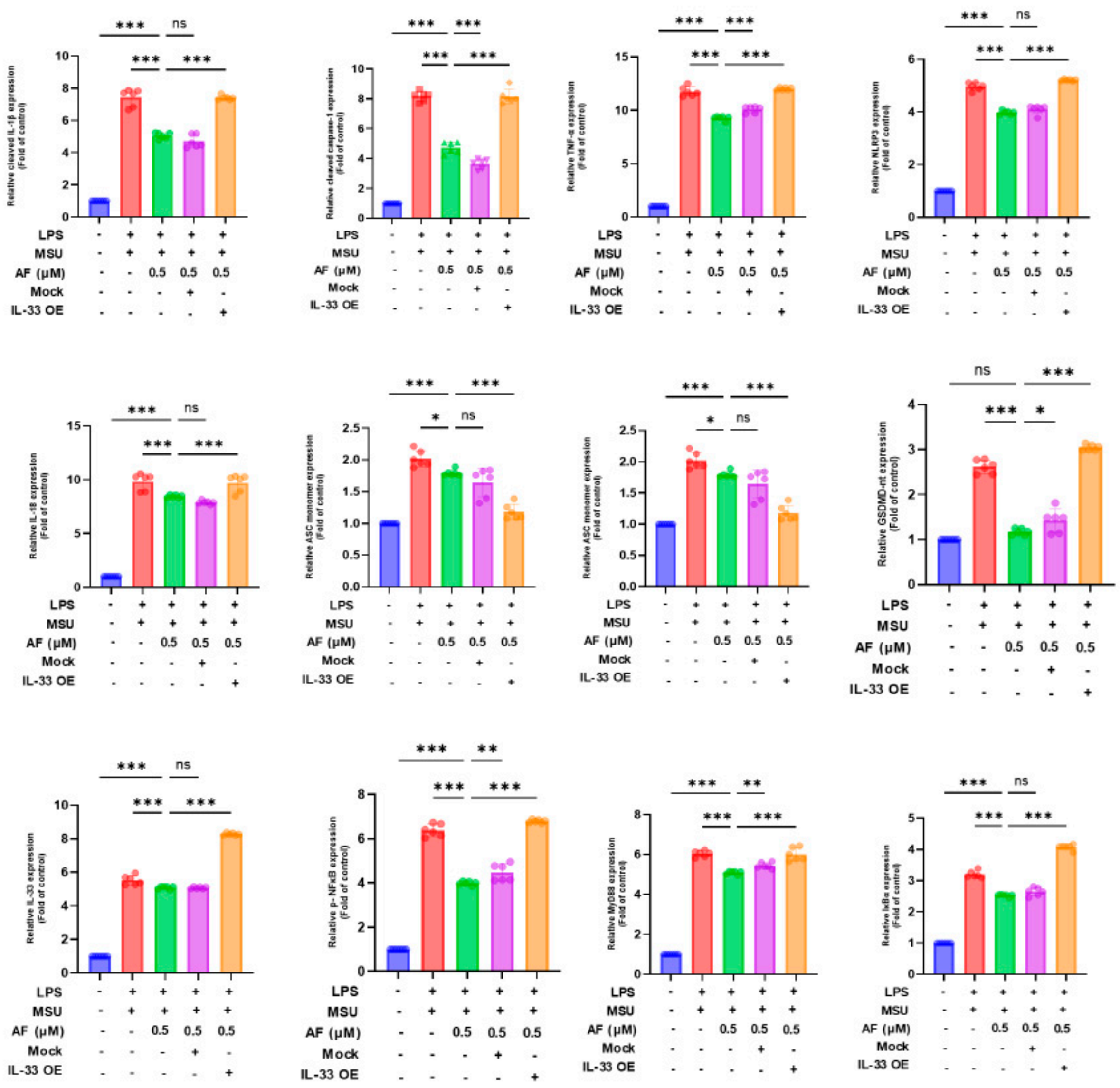
Relative cleaved IL-1β expression (Fold of control)
Relative cleaved caspase-1 expression (Fold of control)
Relative TNF-α expression (Fold of control)
Relative NLRP3 expression (Fold of control)
Relative IL-18 expression (Fold of control)
Relative ASC monomer expression (Fold of control)
Relative GSDMD-nt expression (Fold of control)
Relative IL-33 expression (Fold of control)
Relative p- NFκB expression (Fold of control)
Relative MyD88 expression (Fold of control)
Relative IκBα expression (Fold of control)
LPS
MSU
AF (μM)
Mock
IL-33 OE
0.5
***
**
*
ns

**B**

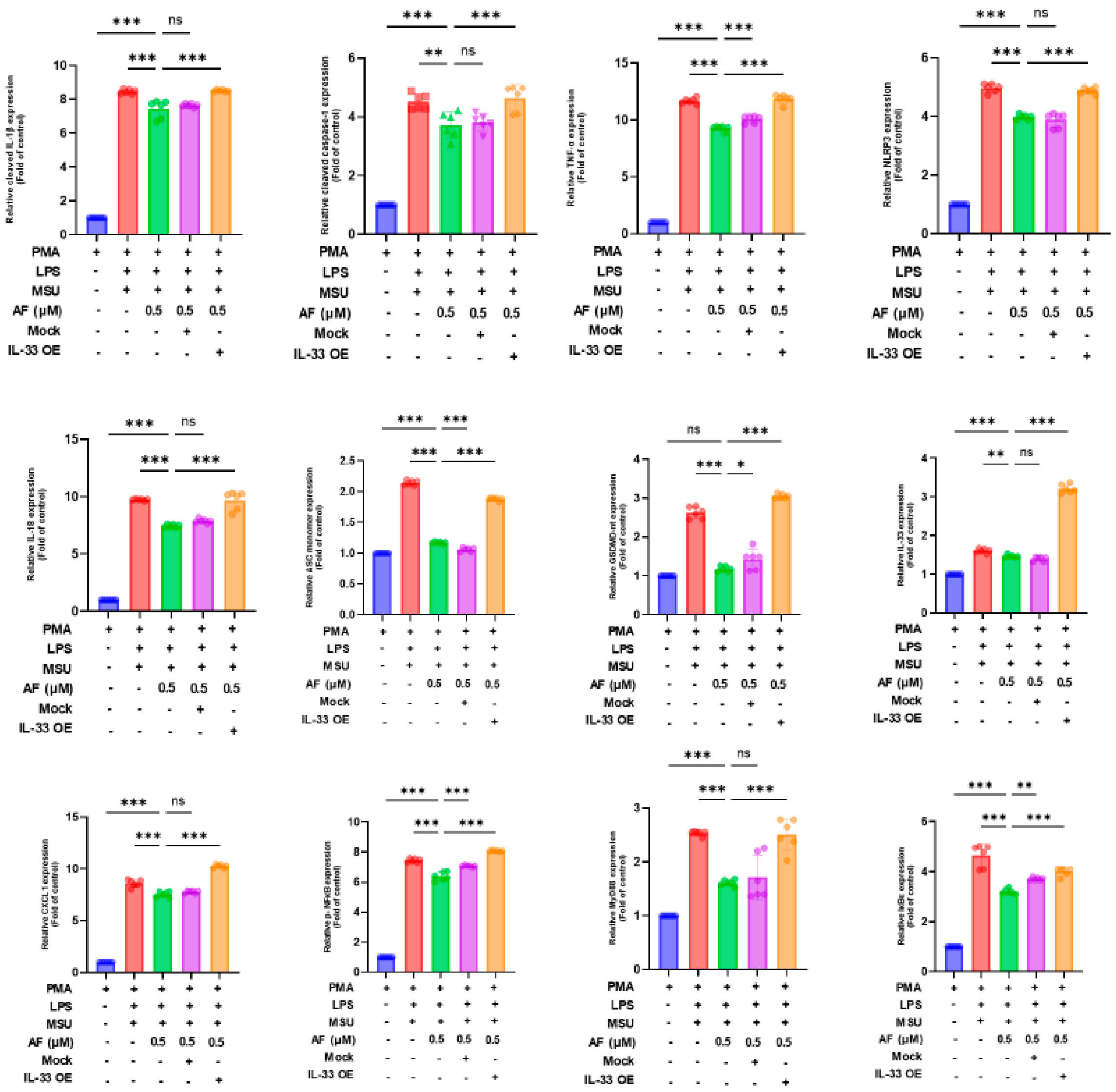


**Supplemental Figure 4. Quantitative analysis of IL-33 overexpression effects in THP-1 and Raw 264.7 cells.**

(A)Densitometric analysis of immunoblot data from Figure 7D, including all evaluated inflammatory markers.
(B) Densitometric analysis of immunoblot data from Figure 7I, including all evaluated inflammatory markers. Data are shown as mean ± SEM from three independent experiments. *P < 0.05, **P < 0.01.

| Gene | Species | Forward primer (5′→3′) | Reverse primer (5′→3′) |
|---|---|---|---|
| Il1b | Mouse | TGAAGAGCCCATCCTCTGTGA | TGCTGATGTACCAGTTGGGG |
| Il18 | Mouse | CGCTTTCTGGCATCTTCTTC | GTCGGCAAGAGGTAGTGACA |
| Cxcl1 | Mouse | CTGGGATTCACCTCAAGAACATC | CAGGGTCAAGGCAAGCCTC |
| Tnf | Mouse | CCCTCACACTCAGATCATCTTCT | GCTACGACGTGGGCTACAG |
| Nlrp3 | Mouse | GTTGTTGTGGAAGCACCTCA | GGTGTGTGAAGATGTTGCAG |
| Il33 | Mouse | GTGACGGTGAGAATGACGAG | TGTCTTCCATGGTGGTGGTG |
| Myd88 | Mouse | GGTCTGTGGGAACCTGTTGT | TCTGAAAGGGATGGCAGAGG |
| Nfkbia | Mouse | CGAGACTCGTGACTGAGGAA | GGTCAGTTGTTGGGGAAGTG |
| Rela | Mouse | CGAGACGACAGCCTTGTAGG | GGGTGCGTCTTAGTGGTATCTG |
| IL1B | Human | GTCAGCCAATCTTCATTGCTC | GTTCATCTCGGAGCCTGTAGT |
| IL18 | Human | CCTGGAATCAGATTACTTTGGAAG | GCAAGAAAGTGAAGTGTGGTCT |
| CXCL1 | Human | CGCTGGCTTAGAACGAAGTC | GAGTGGCTATGACTTCGGTTT |
| TNF | Human | CCAGGGACCTCTCTCTAATCA | TCAGCTTGAGGGTTTGCTAC |
| NLRP3 | Human | GCTGCTCTTCACTGCTATCA | GAGATGAGGCTGACCAGGAA |
| IL33 | Human | TGAGACACCTGGATTCCTGG | CTTCAGGCATCATTTCAAGCC |
| MYD88 | Human | AGGAGGAAATGGCTCAGGTG | TCCAGGATGGTGGTAGAGGA |
| NFKBIA | Human | ATCAGCCCTCATTTTGTTGC | ACACATGGCAGACGCTAGGA |
| RELA | Human | AGAGGGAGCAGAGGAGCAAT | AGCTGTCAGTGTCAGGGTGA |

**Supplementary Table 1. Primer sequences used for qPCR analysis.**

**Supplementary Methods**

MTT cell viability assay

Reagents and general conditions.
3-(4,5-Dimethylthiazol-2-yl)-2,5-diphenyltetrazolium bromide (MTT) was prepared as a 5 mg/mL stock in PBS, sterile-filtered (0.22 µm), aliquoted, and stored at 4 °C protected from light. Auranofin was dissolved in DMSO and diluted in culture medium immediately before use (final DMSO ≤ 0.1% v/v in all wells, including vehicle controls). Unless otherwise stated, assays were performed in flat-bottom 96-well plates (total volume 100 µL/well) at 37 °C, 5% $CO_2$.

THP-1-derived macrophages.
THP-1 cells were differentiated with PMA as described in the main Methods. After the 24 h rest in PMA-free medium, cells were washed and reseeded at $1.5 \times 10^4$ cells/well in 96-well plates. Cells were pre-treated with Auranofin **(**0.001–5 µM**)** for 2 h, then stimulated with LPS (100 ng/mL, 2 h) followed by MSU (200 µg/mL) as indicated. Total exposure time to Auranofin was 24 h (matching the stimulation window). For each plate, vehicle (DMSO ≤ 0.1%) and positive cytotoxicity control **(**e.g., 0.1% Triton X-100**,** 30 min before MTT) were included. To control for any non-enzymatic dye reduction or optical interference, blank wells containing complete medium ± Auranofin and/or MSU without cells were processed in parallel.

Mouse bone marrow–derived macrophages (BMDMs).
Day-7 BMDMs were plated at $2.5 \times 10^4$ cells/well and treated in an identical schedule: Auranofin pre-treatment (0.005–0.5 µM, 2 h), LPS priming (100 ng/mL, 4 h), and MSU challenge (100 µg/mL), with a total Auranofin exposure of 24 h.

MTT reaction and reading
At the end of treatments, MTT working solution was added to each well to a final concentration of 0.5 mg/mL (add 10 µL of 5 mg/mL stock to 100 µL culture). Plates were incubated for 4 h at 37 °C in the dark until purple formazan crystals were apparent. Supernatants were carefully removed and formazan was solubilised by adding 100 µL DMSO (or acidified isopropanol) to each well and shaking for 10 min at room temperature. Absorbance was measured at 570 nm with a reference wavelength of 630–690 nm using a microplate reader.

Data processing

Background absorbance from blank wells (no cells) was subtracted from all readings. Cell viability was calculated relative to the vehicle control according to:

$$\%\,\text{Viability} = (\text{A\_vehicle} - \text{A\_blank}) / (\text{A\_treated} - \text{A\_blank}) \times 100$$

Notes and controls

MSU crystals can cause light scatter; therefore MSU-only blanks (no cells) were included for background correction.

Auranofin is light-sensitive; all steps involving MTT and Auranofin were conducted protected from light.

Concentrations used here Auranofin **(**0.001–5 µM**)** match those in the main experiments to directly relate cytotoxicity to functional readouts.
